# Effectiveness of acupuncture in the governor vessel and Yangming meridian for the treatment of acute ischemic stroke: A systematic review and network meta-analysis

**DOI:** 10.1371/journal.pone.0300242

**Published:** 2024-04-16

**Authors:** Yingqi Xu, Xiuzhen Xie, Pingping Su, Jiashan Wang, Xinxin Luo, Jianli Niu, Zhuqing Jin

**Affiliations:** 1 The Second School of Clinical Medicine, Zhejiang Chinese Medical University, Hangzhou, China; 2 The First School of Clinical Medicine, Zhejiang Chinese Medical University, Hangzhou, China; 3 The Third School of Clinical Medicine, Zhejiang Chinese Medical University, Hangzhou, China; 4 Office of Human Research, Memorial Healthcare System, Hollywood, Florida, United States of America; 5 School of Basic Medical Sciences, Zhejiang Chinese Medical University, Hangzhou, China; China Medical University, TAIWAN

## Abstract

**Background:**

Acupuncture of the governor vessel and Yangming meridian are widely used in the treatment of acute ischemic stroke (AIS). However, the optimal meridian for acupuncture in the treatment of AIS remains uncertain.

**Purpose:**

This network meta-analysis study aimed to compare the clinical effectiveness of acupuncture at governor vessel and Yangming meridian in the treatment of AIS.

**Methods:**

All relevant studies published in CNKI, WANFANG, VIP, Sinomed, Cochrane Library, Web of Science, Pub Med, and Embase before January 13, 2024 were systematically retrieved. The two researchers independently screened the studies and extracted the data. Cochrane ROB tool was used to evaluate the quality of the studies, and Stata 14.0 software was used to conduct a network meta-analysis of neurological deficit score, activities of daily living (ADL), clinical effective rate and Fugl-meyer motor function evaluation (FMA).

**Results:**

A total of 401 studies were obtained, and 17 studies met the inclusion criteria. The surface under the cumulative ranking curve (SUCRA) values of the four outcome indexes were all ranked by “Governor vessel acupuncture + Conventional neurology treatment(GVAc+CT) > Yangming meridian acupuncture + Conventional neurology treatment(YMAc+CT) > Conventional neurology treatment (CT)”. Compared to YMAc+CT and CT, GVAc+CT had the best effect in reducing the degree of neurological deficit score (SMD = -0.72, 95%CI = [-1.22,-0.21] and SMD = -1.07,95%CI = [-1.45,-0.69], respectively) and promoting the recovery of ADL((SMD = 0.59,95%CI = [0.31,0.88] and SMD = 0.96,95%CI = [0.70,1.21], respectively). Compared to CT, GVAc+CT also had a better clinical effective rate in the treatment of AIS (RR = 1.14,95%CI = [1.04,1.25]).

**Conclusions:**

Governor vessel acupuncture combined with conventional neurology treatment has the best effect in reducing the degree of neurological deficit score and promoting the recovery of ADL in AIS patientscompared to YMAc+CT and CT. Governor Vessel acupuncture is the most preferable acupoint scheme for clinical acupuncture treatment of AIS.

## Introduction

Acute ischemic stroke (AIS) is a medical emergency in which the arteries supplying blood to a specific region of the brain are narrowed or blocked, resulting in the interruption of local cerebral microcirculation in the cerebral area and the initiation of an ischemic cascade response that eventually leads to brain cell damage [1, 2]. In recent years, a large number of studies have found that acupuncture treatment for AIS can enhance nerve regeneration, improve cerebral blood flow, reduce inflammatory damage and neuroexcitotoxicity and apoptosis caused by cerebral ischemia, thus promoting the recovery of brain function after AIS and effectively improving the prognosis of patients with sudden cerebrovascular accidents [3, 4]. At the same time, the earlier application of acupuncture treatment is associated with greater benefits for the recovery of neurological function in patients with AIS [5]. Compared with drugs, surgery and other treatment methods, acupuncture is more convenient, cost-effective, and has fewer side effects. The World Health Organization has recommended acupuncture as a viable alternative and complementary strategy for stroke treatment [6].

In animal experiments, the governor vessel electroacupuncture has been shown to promote the cortical somatosensory evoked potential (SEP) recovery and reduce the volume of cerebral infarction after cerebral ischemia, which is stronger than body acupuncture [7]. Zhou F [8] demonstrated by animal experiments that acupuncture at various meridians improved cerebral blood flow and recovery of patients with varying degrees of damage by cerebral ischemia, indicating the importance of the selection of different meridians and acupoints in the treatment of AIS with acupuncture. WangSL found that both acupuncture on the governor vessel acupuncture and yangming meridian could up-regulate the mRNA expression of MAP-2 and NF-L in the brain tissue of rats, thereby improving brain plasticity and promoting neuronal regeneration, with the governor vessel acupuncture group exhibiting a more significant effect [9]. In a study by Pan Jiang, electroacupuncture at the governor vessel was found to enhance the expression of NGF in the cerebral infarction area, leading to a reduction in the volume of cerebral infarction and resisting nerve damage [10]. Furthermore, the governor vessel group showed a higher NGF expression in the infarct area compared to the Yangming meridian group. These animal experiments shed light on crucial mechanisms underlying the difference in clinical efficacy between the two acupuncture approaches.

**Fig 1:**
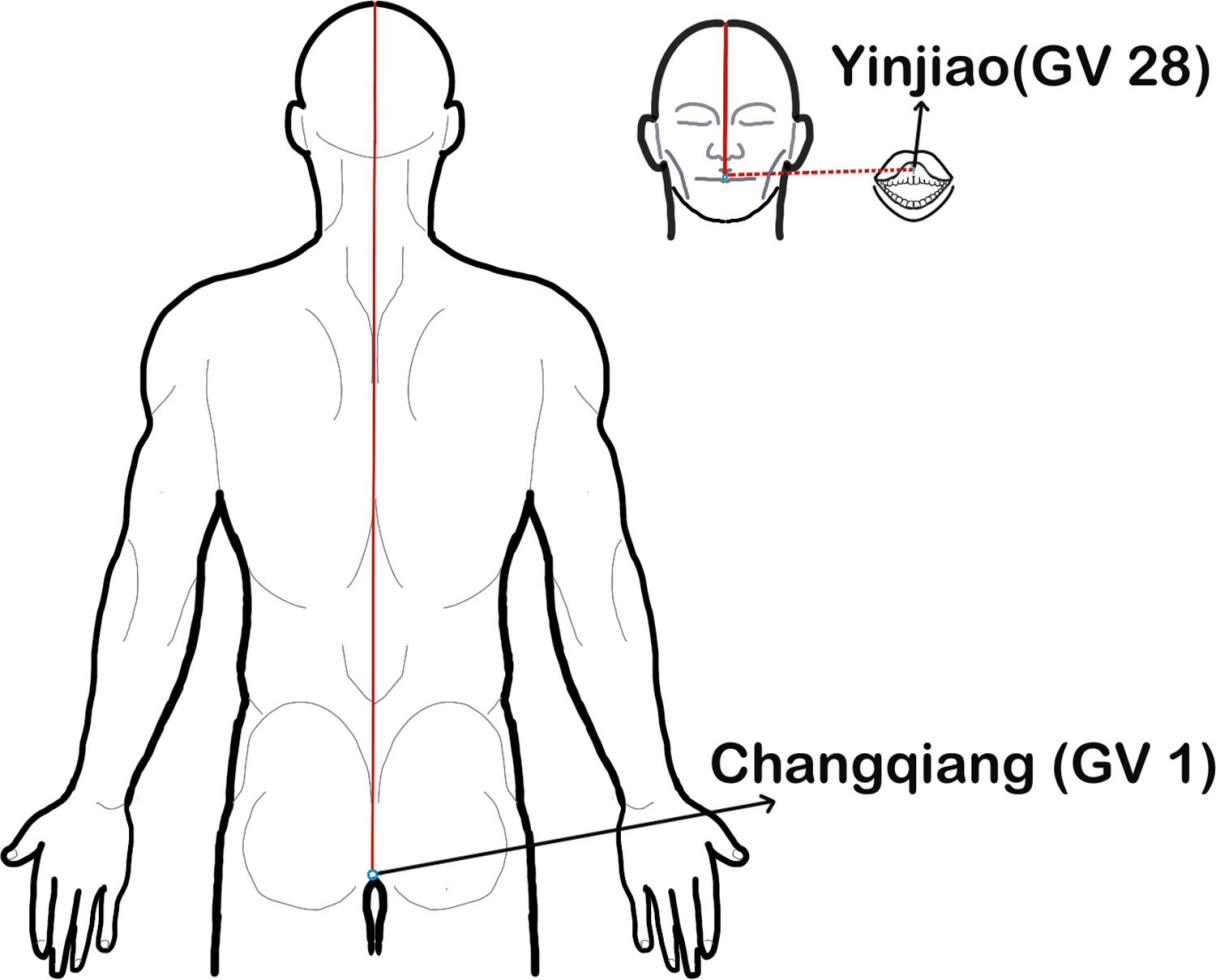
Main trunk of governor vessel acupoints. **Note:** In order to show more clearly the main pathways of the three meridians running through the muscles on the body surface, the red solid lines in only show the main pathways connected by each meridian point.

**Fig 2:**
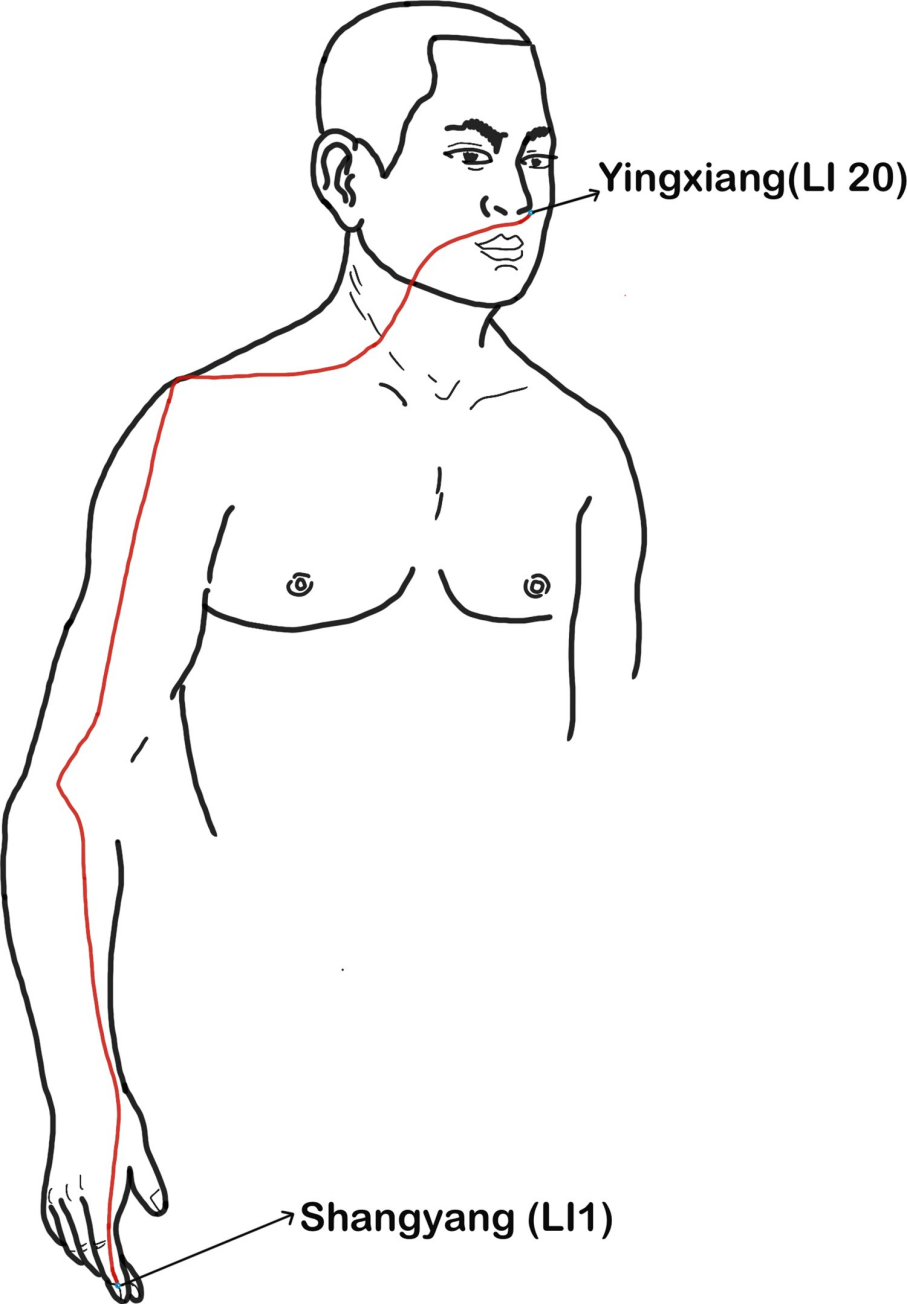
Main trunk of Yangming Large Intestine Meridian of Hand acupoints. **Note:** In order to show more clearly the main pathways of the three meridians running through the muscles on the body surface, the red solid lines in only show the main pathways connected by each meridian point.

**Fig 3:**
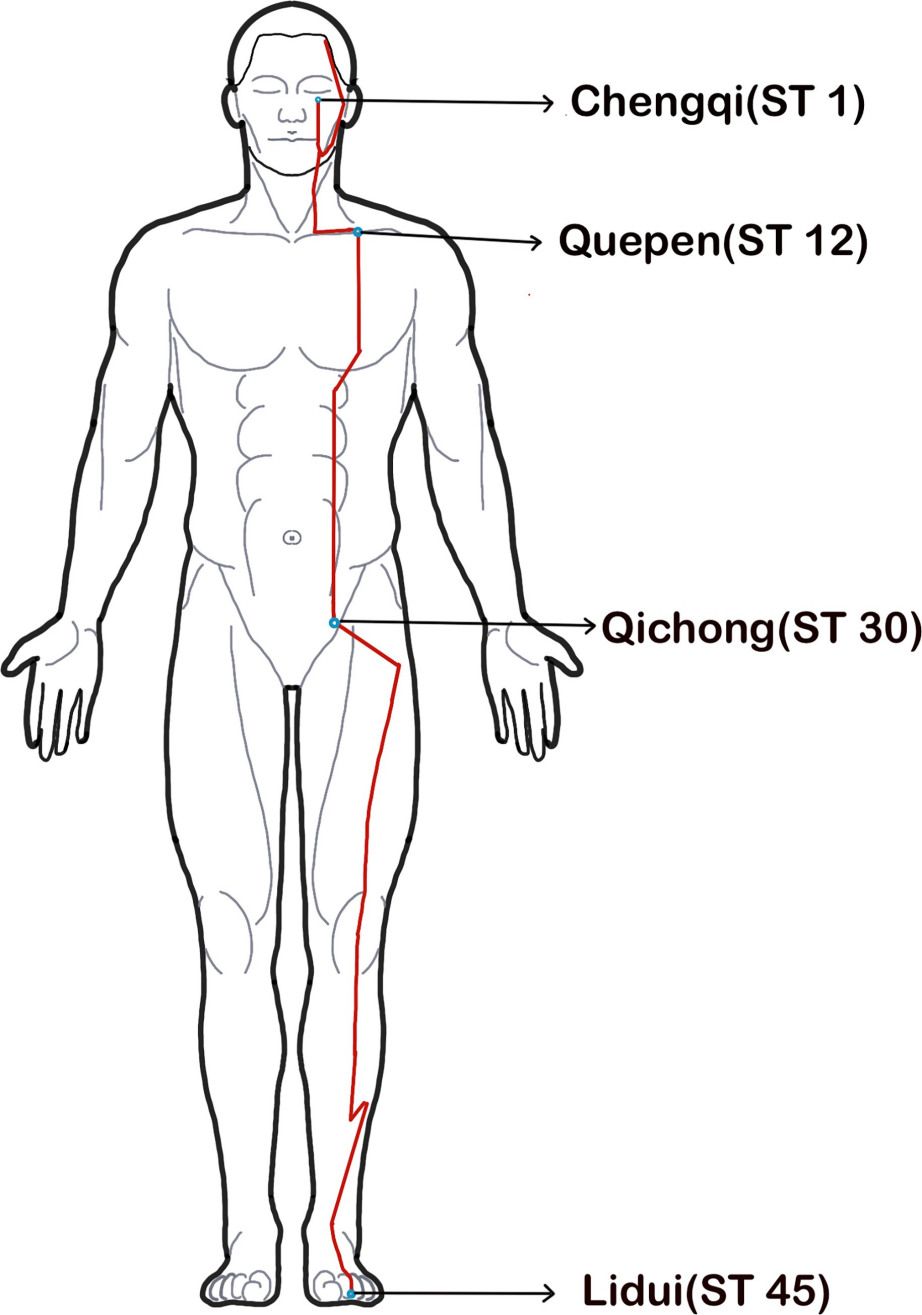
Main trunk of Yangming Stomach Meridian of Foot acupoints. **Note:** In order to show more clearly the main pathways of the three meridians running through the muscles on the body surface, the red solid lines in only show the main pathways connected by each meridian point.

The main trunk of the governor vessel starts from Changqiang (GV1, the midpoint of the line between the end of the coccyx and the anus) along the posterior midline of the body and ends at the Yinjiao (GV28, the junction of the upper lip frenulum and gingiva), and is closely related to the brain and spinal cord (Fig 1). The Yangming meridian includes Yangming Large Intestine Meridian of Hand and Yangming Stomach Meridian of Foot: the Yangming Large Intestine Meridian of Hand (Fig 2) mainly follows the radial side of the upper limb from Shangyang (LI1) to the neck and face, finally stops at Yingxiang (LI20); The straight trunk of the Yangming Stomach Meridian of Foot (Fig 3) starts from the Chengqi (ST1) and runs along the neck to Quepen (ST12), then it goes down to the Qichong (ST30) located at the inguinal artery along the front of the thigh and the outside of the calf until it reaches the Lidui (ST44). Unilateral upper and lower limb dysfunctions are the most common symptoms of AIS [11]. Therefore, Yangming meridian acupuncture is also commonly used in the treatment of AIS.

According to the current basic and clinical research progress of acupuncture in the treatment of cerebral ischemia, we planned to conduct a systematic review by integrating the existing clinical studies and using the network Meta-analysis method to compare the therapeutic effect of acupuncture at governor vessel and Yangming meridian in the treatment of AIS, so as to provide evidence-based medical basis for the selection of acupoints of meridians in the treatment of AIS.

## Materials and methods

This network meta-analysis was not registered on the website.

### Search strategy

This study was conducted according to the Preferred Reporting Items for Systematic Review and Network Meta-analysis (PRISMA NMA) checklist (S1 File). Literature published in CNKI, WANFANG, VIP, sinomed, Cochrane Library, Web of Science, Pub Med, and Embase was systematically searched using the following search terms (S2 File): (Acute ischemic stroke OR Acute brain Infarction OR Acute Ischemia Apoplexy OR Acute cerebral infarction OR Acute cerebral embolism OR Acute Ischemic Apoplexy OR Acute brain Ischemia OR AIS OR ACI) AND (governor vessel OR governor meridian OR du meridian OR yangming meridian) AND (acupuncture OR electropuncture). The range of publication dates were from their establishment of the database to January 13, 2024. All published clinical randomized controlled trials (RCTs) of acupuncture at acupoints of governor vessel or Yangming meridian in the treatment of AIS, including conference and academic literature were included without language restrictions.

### Eligibility criteria

#### Inclusion criteria

1. Study types: clinical RCT studies, published language unlimited, including conference literature;
2. Study population: (a) Patients diagnosed with AIS following the “Guidelines for diagnosis and treatment of Acute Ischemic Stroke in China 2014” [12] and were confirmed by imaging (including CT, MRI, etc.). (b) The onset of the AIS was less than 14 days;
3. Intervention measures: (a) The control group was treated with conventional neurology treatment (CT), including thrombolysis, antiplatelet, anticoagulation, defibrination, volume expansion, blood lipid regulation, neuroprotection, and rehabilitation training. (b) In addition to the treatments in the control group, the treatment group added acupuncture at the main acupoints of governor vessel or Yangming Meridian as a means of treatment;
4. Outcome measures: Neurological deficit score, Activities of daily living (ADL), Clinical effective rate, and Fugl-meyer motor function evaluation (FMA).

### Exclusion criteria

1. Study types: animal experiments, reviews, case reports, etc.;
2. Study population: The onset time is not clear;
3. Intervention measures: (a) The control group was treated with sham acupuncture, traditional Chinese medicine, etc.; (b) In the experimental group, the main acupuncture points were selected by other meridians or with massage, Chinese medicine as a means of treatment;
4. Insufficient original data, data duplication, omission, etc. in clinical research literature.

### Study screening and data extraction

Two independent reviewers (XYQ and XXZ) used the NoteExpress software to strictly search, screen, and manage the literature. The following data were extracted and stored in a spreadsheet database: (1) the first author of the study and year of publication; (2) patient information (Disease duration, number, gender and mean age); (3) intervention information (Intervention and control measures and treatment duration); (4) outcome indicators; (5)adverse events. The results of literature screening and data extraction were cross-checked. Discrepancies in the data extraction were resolved by the discussion with the third reviewer (JZQ).

### Certainty of evidence assessment

Another two independent reviewers (SPP and WJS) evaluated the quality of the studies based on the quality assessment scale in the Cochrane Systematic Assessment Manual [13] using RevMan(cochrane website, 5.3.5) software in seven aspects: randomization method, allocation concealment, blinding (patient, operator), data integrity, selective data, and other biases, and finally "high risk", "low risk", and "unknown risk" were obtained. The quality of four outcome indicators was evaluated according to the results of network Meta-analysis from five aspects: research limitations, inconsistency, indirectness, precision and publication bias, based on the GRADE framework (Grading of Recommendation, Assessment, Development, and Evaluation) in the Cochrane handbook [13]. Finally, four grades of high, medium, low and very low were obtained, and a summary table was made.

### Statistical analysis

A random-effect model was used for the network meta-analysis.Each outcome measure was assessed using validated scales: Neurological deficit score was evaluated by Composite Score Scale (CSS) and National Institute of Health stroke scale (NIHSS). ADL was assessed by Modified Barthel Index (MBI) and Barthel Index (BI). FMA was assessed by Fugl-Meyer assessment scale. The number of points reduction to be considered effective varies per study, and clinical effective rate was obtained according to the criteria of each study. The time point of the outcome indicators that were investigated was the earliest result following the completion of the study treatment course, and the difference before and after the intervention of each outcome index was selected for analysis. Neurological deficit score, ADL and FMA were continuous variables, due to different measurement methods, the standardized mean difference (SMD) was used to eliminate the effect. Clinical effective rate was a dichotomous variable and the effect size was expressed as a relative risk (RR). If a closed loop was formed, global inconsistency test was carried out to determine whether the consistency model was used, P > 0.05 indicates that there was no global inconsistency, and the consistency model can be used. At the same time, the loop inconsistency test was carried out, node-splitting analysis and inconsistency factors were used to test inconsistency for closed-loop indirect comparison. If the lower limit of the 95% confidence interval contained or near 0, it was considered that there was no loop inconsistency, and the direct and indirect comparative evidence were considered consistent. The STATA (StataCorp LLC, 14.0, "mvmeta" and "network" packages) was used to draw evidence network and to more intuitively observe the relationship between interventions. League tables were used to compare different interventions. The SUCRA was used to evaluate the ranking probability value of each intervention and to summarize the best choice of meridian acupuncture for AIS. In addition, subgroup analyses were performed post hoc with the use of RevMan 5.3 software. The 95% confidence interval indicates the size of the statistical effect. If there was no significant heterogeneity among the studies (P≥0.1, I^2^<50%), a fixed-effects model was used, otherwise using a random-effects model (P<0.1, I^2^≥50%). In order to evaluate the stability of the results, the source of heterogeneity was explored by subgroup analysis. A correction-compare funnel diagram was used to evaluate the publication bias of each outcome index of the studies.

## Results

### Study inclusion

**Fig 4:**
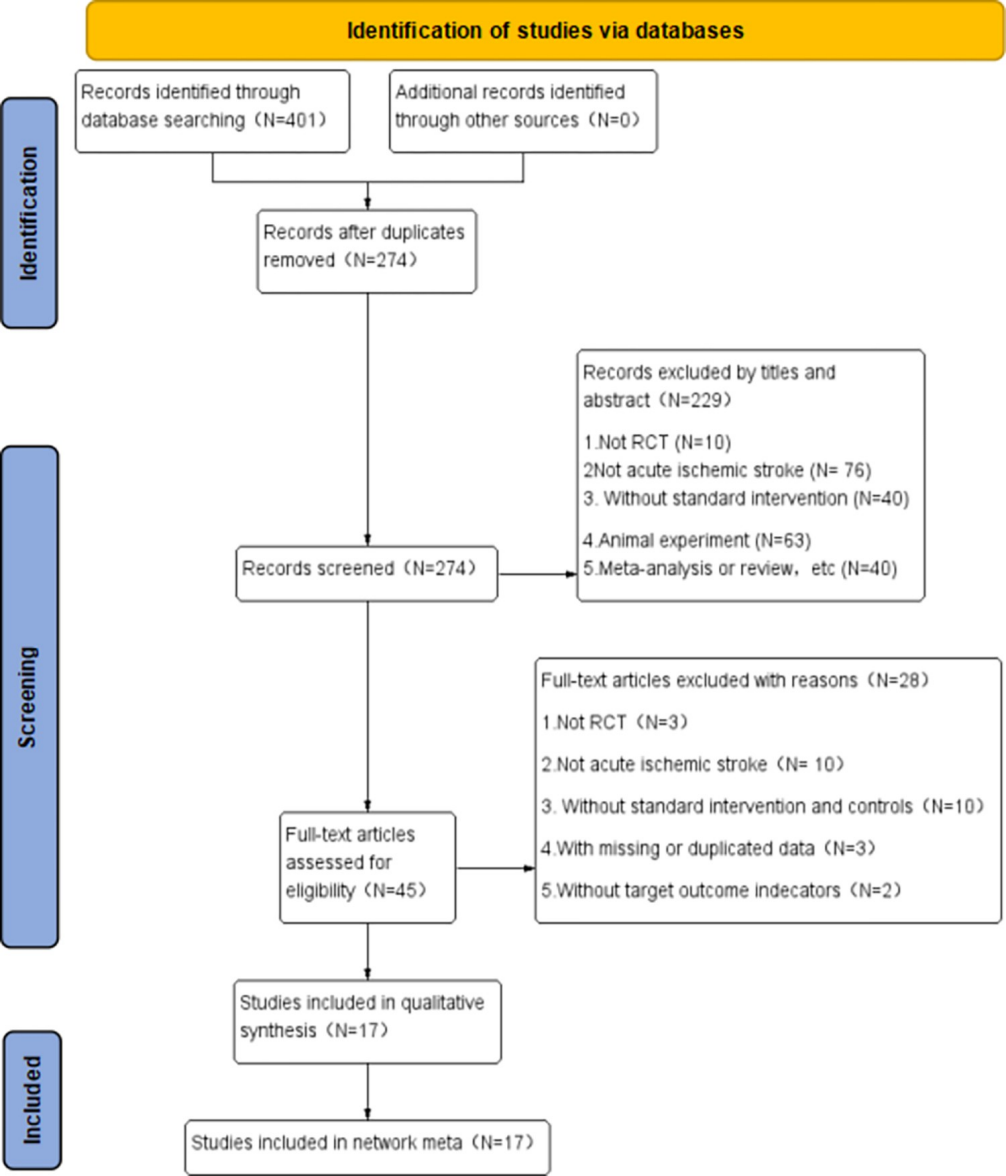
PRISMA flowchart.

The search yielded 401 articles and 274 were obtained after subtracting duplicates. After reviewing the title, abstract and full text, 17 papers were finally included in meta-analysis. Fig 4 shows the retrieval process.

### Study characteristics

A total of 1269 patients with AIS were included in the 17 RCTs, with 15 [14–22, 24–27, 29, 30] two-arm studies and 2 [23, 28] three-arm studies (Table 1). Most of the included treatment groups retained the needle for 20–30 min after deqi, and the course of treatment was 10–40 days. Among them, 5 [17, 24, 27, 29, 30] studies used electroacupuncture at a frequency of 1-20HZ, and the intensity was based on patient tolerance.

**Table 1:** Baseline characteristics of the included studies.

Study	Course of disease	acupuncture manipulation;stimulation frequency	No. of participants (male/female);age (years)	Therapy			Conventional neurological treatment drugs	Treatment time	Outcome
				T1	T2	C			
Wei et al.(2023) [14]	<4.5h	non-EA	40(23/17)	GVAc+CT		CT	rt-PA	4w	①②③④
		NA	(52.13±6.70)						
Li et al.(2022) [15]	0.8~4.1h	non-EA	100(58/42)		YMAc+CT	CT	N	4w	①②③④
		180–200 times/min	(63.95±6.19)						
Ma et al.(2021) [16]	<2d	non-EA	83(47/36)	GVAc+CT		CT	N	4w	①②④
		NA	(61.58±5.57)						
Wang et al.(2021) [17]	1~14d	EA	73(40/33)		YMAc+CT	CT	Aspirin, Clopidogrel, Atorvastatin calcium, Troxerutin and Cerebroprotein Hydrolysate Injection	14d	①②④
		20HZ	(50.52±13.49)						
Liu(2020) [18]	6~33h	non-EA	126(71/55)	GVAc+CT	YMAc+CT		N	28d	①②③
		NA	(70.6±7.3)						
Hua et al.(2019) [19]	24h~2w	non-EA	100(50/50)	GVAc+CT		CT	Aspirin	14d	③
		NA	(63±10.6)						
Li(2018) [20]	<24 h	non-EA	37(21/16)	GVAc+CT		CT	N	4w	①②
		NA	(57.95±8.5)						
ZhangL et al.(2017) [21]	<48h	non-EA	37(22/15)	GVAc+CT		CT	N	4w	①③
		NA	(65.73±9.01)						
Cai et al.(2016) [22]	4-5h	non-EA	60(31/29)	GVAc+CT		CT	Ozagrel Sodium for Injection	2w	①③
		NA	(53.60±10.91)						
Li et al.(2016) [23]	<2w	non-EA	90(58/32)	GVAc+CT	YMAc+CT	CT	Edaravone, Aspirin	10d	①②③
		NA	(62.54±10.27)						
Ma et al.(2016) [24]	5~45h	EA	78(40/38)	GVAc+CT	YMAc+CT		N	24d	①②③
		NA	(61.48±4.41)						
Yu(2014) [25]	1.79d~6.39d	non-EA	65(44/21)	GVAc+CT		CT	N	14d	②③④
		≤200 times/min	(61.82±4.89)						
Tan et al.(2013) [26]	<72h	non-EA	94(51/43)	GVAc+CT		CT	N	10d	①③
		NA	(61.47±12.09)						
Huang et al.(2012) [27]	4h~6d	EA	58(28/30)		YMAc+CT	CT	N	14d	①②
		20HZ	(67.64±10.48)						
Ge(2009) [28]	7h~3d	non-EA	90(53/37)	GVAc+CT	YMAc+CT	CT	Ozagrel Sodium and Sodium Chloride Injection, Cerebroprotein Hydrolysate for Injection	21d	①②③
		NA	(60.16±10.88)						
ZhangX et al.(2008) [29]	4h~6d	EA	58(30/28)		YMAc+CT	CT	Cinepazide Maleate Injection, Aspirin, Piracetam	2w	①②④
		20HZ	(67.64±10.48)						
ZhangSJ et al.(2007) [30]	<5d	EA	80(56/24)	GVAc+CT		CT	N	15d	①③
		1HZ	(61.85±11.10)						

**Note:** T1, treatment group 1; T2, treatment group 2; C, control treatment group; GVAc, governor vessel acupuncture; YMAc, Yangming meridian acupuncture; CT, conventional neurology treatment; EA,electroacupuncture; non-EA, non-electroacupuncture; N, Only the use of conventional neurological treatment was mentioned, including thrombolysis, antiplatelet, anticoagulation, fibrin reduction, volume expansion, lipid regulation, neuroprotection, and rehabilitation. No specific medications were mentioned. ①: Neurological deficit score; ②: Activities of daily living (ADL); ③: Clinical effective rate;④: Fugl-meyer motor function evaluation(FMA).

### Analysis of methodological quality evaluation

**Fig 5:**
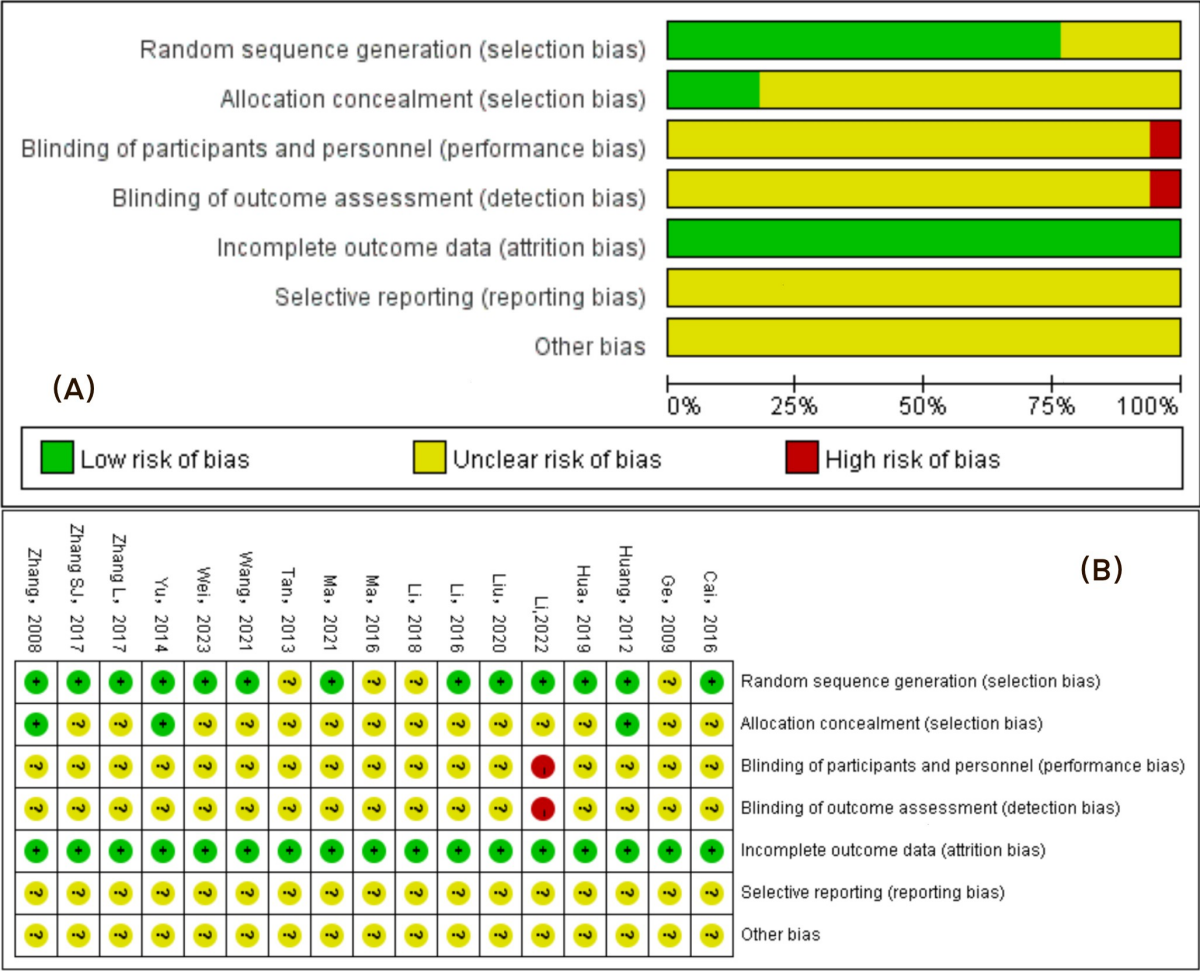
Methodologic quality assessment of the risk of bias. **Note:** Low risk, unclear risk and high risk are indicated by the symbols “+”,“?”, and “-” respectively. (A) Risk of bias graph. (B)The bias risk and the quality evaluation results of each included study by the Cochrane risk of bias tool.

The quality of the included trials was assessed according to the bias risk tool of the Cochrane Collaboration. ① In terms of random methods, 13 [14–19, 21–23, 25, 27, 29, 30] studies applied random number table method, which could be rated as low risk, and 4 [20, 24, 26, 28] studies only mentioned randomness and were rated as unknown risks; ② In terms of allocation hiding,3 [25, 27, 29] articles with random envelope allocation were rated as low risk, and the rest that were not mentioned were rated as unknown risk; ③ In terms of blinded protocol (patient and operator), 1 [15] study was clearly non-blinded and rated as high risk, the rest that did not mention the blinded protocol were rated as unknown risk; ④ In terms of ending data integrity, 4 [16, 20, 21, 25] studies reported the number of data dislodgements and the reasons for them and were rated as low risk, while the rest that did not have incomplete data were rated as low risk; ⑤ In terms of selective reporting and other bias, all the studies that did not mention the relevant information and were rated as unknown risks (Fig 5).

### Evidence network

**Fig 6:**
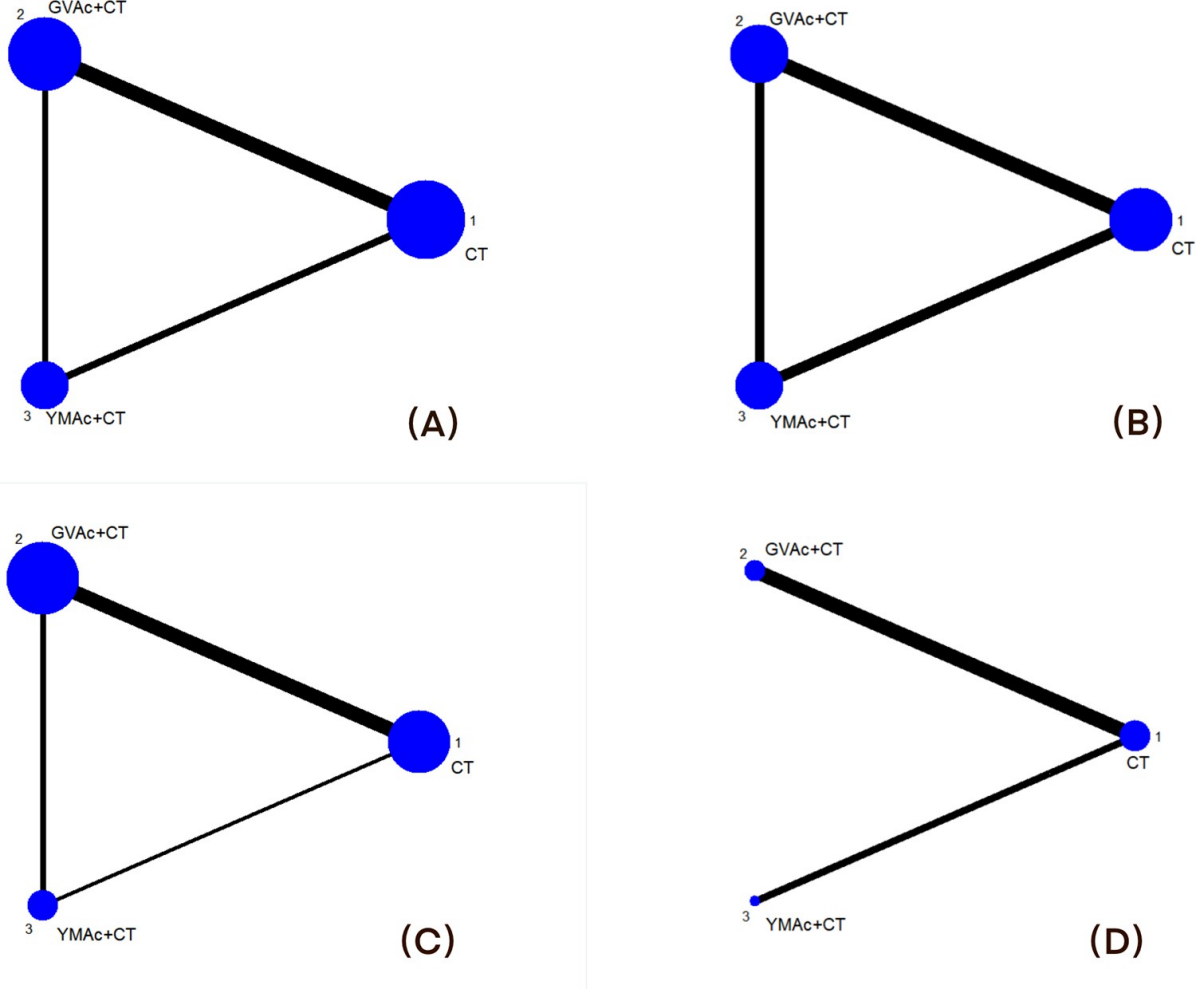
Evidence network. **Note:** GVAc = governor vessel acupuncture; YMAc = Yangming meridian acupuncture; CT = conventional neurology treatment; (A)Neurological deficit score; (B): ADL; (C): Clinical effective rate; (D): FMA.

Fig 6 shows the network diagram of interventions under different outcome measures. In the network map, three interventions(GVAc+CT, YMAc+CT and CT) were considered as nodes. Pairwise comparisons between interventions served as lines of the network map. The thickness of the black line is the number of comparative studies, the blue dots are the interventions and the sizes of the blue dots are the number of samples participating in the intervention. The thicker of the black line and the larger of the blue circle correspond to the comparison study and sample size. 15 [14–18, 20–24, 26–30] studies reported neurological deficit score, with closed loop formation; 12 [14–18, 20, 23–25, 27–29] studies reported ADL, with closed loop formation; 12 [14–15, 18, 19, 21–26, 28, 30] studies reported clinical effective rate, with closed loop formation and 6 [14–17, 25, 29] studies reported FMA, all of which were indirect evidence without closed loop formation, and no inconsistency detection was required.

### Inconsistency test

There was a closed loop that was formed in the evidence network of three outcome indicators (ADL, Clinical effective rate and Neurological deficit score), namely “GVAc+CT—YMAc+CT—CT”. P values of neurological deficit score (P = 0.7567), ADL (P = 0.4483) and clinical effective rate (P = 0.8067) were all > 0.05, suggesting that there is a good overall consistency between the comparisons. The results of loop inconsistency test showed that the inconsistency factor (IF) values of neurological deficit score closed-loop (IF = 0.274,95%CI = [0.00,1.30]), clinical effective rate (IF = 0.051,95%CI = [0.00,0.34]) closed-loop and ADL (IF = 0.286,95%CI = [0.00,0.86]) closed-loo were close to 0, and the lower limit of 95% CI were 0, suggesting that there is a good consistency between direct comparison and indirect comparison among the intervention measures and the result of network meta-analysis is reliable. FMA did not form a closed loop and no inconsistency test was performed.

### Network meta-analysis

#### Neurological deficit score

As shown in Table 2, GVAc+CT (SMD = -0.72, 95%CI = [-1.22,-0.21], low quality evidence / SMD = -1.07,95%CI = [-1.45,-0.69], low quality evidence) was more effective than YMAc + CT and CT in reducing neurological deficit score. The difference were statistically significant (p < 0.05). Other comparative differences were not statistically significant.

**Table 2:** Network meta-analysis of neurological deficit score (SMD and 95%CI).

GVAc+CT		
**-0.72(-1.22,-0.21) ⨁⨁◯◯** ^**1,2,4**^	YMAc+CT	
**-1.07(-1.45,-0.69) ⨁⨁◯◯** ^**1,2**^	-0.35(-0.84,0.13) **⨁◯◯◯**^**1,2,4**^	CT

**Note:** 1: limitations of the study due to blinding or lack of allocation concealment (-1), 2. Indirect comparison in evidence (-1), 3. Global or ring inconsistency leads to (-1), 4. Imprecision due to too small sample size (< 300) or too wide confidence (-1). ⨁◯◯◯/◯◯◯◯ = very low quality evidence, ⨁⨁◯◯ = low quality evidence. (Same comment for the rest of the Tables 3–5.). Comparisons should be read from left to right. Estimates of statistical significances are shown in bold (P < 0.05), and 95% CI must not overlap zero. Negative values indicate better effect of the intervention on the left compared to the right.

#### ADL

As shown in Table 3, GVAc+CT (SMD = 0.59,95%CI = [0.31,0.88], low quality evidence) had a better effect in enhancing ADL of patients than YMAc+CT. GVAc+CT(SMD = 0.96,95%CI = [0.70,1.21], low quality evidence) and YMAc+CT (SMD = 0.36,95%CI = [0.08,0.64], low quality evidence) had better effect in enhancing ADL, compared with CT. All differences were statistically significant (p <0.05).

**Table 3:** Network meta-analysis of ADL (SMD and 95%CI).

GVAc+CT		
**0.59(0.31,0.88) ⨁⨁◯◯** ^**1,2**^	YMAc+CT	
**0.96(0.70,1.21) ⨁⨁◯◯** ^**1,2**^	**0.36(0.08,0.64) ⨁⨁◯◯** ^**1,2**^	CT

**Note:** Comparisons should be read from left to right. Estimates of statistical significances are shown in bold (P < 0.05), and 95% CI must not overlap zero. Positive values indicate better effect of the intervention on the left compared to the right.(Tables 3,5)

#### Clinical effective rate

As shown in Table 4, GVAc+CT(RR = 1.14,95%CI = [1.04,1.25], low quality evidence) had a better effect in improving clinical effective rate, compared with CT. Other comparative differences were not statistically.

**Table 4:** Network meta-analysis of clinical effective rate(RR and 95%CI).

GVAc+CT		
1.12(0.98,1.27) **⨁◯◯◯**^**1,2,4**^	YMAc+CT	
**1.14(1.04,1.25) ⨁⨁◯◯** ^**1,2**^	1.02(0.88,1.18) **⨁◯◯◯**^**1,2,4**^	CT

**Note:** Comparisons should be read from left to right. Estimates of statistical significances are shown in bold (P < 0.05), and 95% CI must not overlap 1. The RR > 1 indicate better effect of the intervention on the left compared to the right.(Table 4)

#### FMA

As shown in Table 5, there was no statistical significance between the comparisons (p < 0.05), and the evidence related to this outcome indicator was very low quality evidence.

**Table 5:** Network meta-analysis of FMA(SMD and 95%CI).

GVAc+CT		
0.31 (-0.97,1.59) **◯◯◯◯**^**1,2,3,4**^	YMAc+CT	
0.41 (-0.33,1.51) **◯◯◯◯**^**1,2,3,4**^	0.10 (-0.95,1.15) **◯◯◯◯**^**1,2,3,4**^	CT

### SUCRA

#### Neurological deficit score

**Fig 7:**
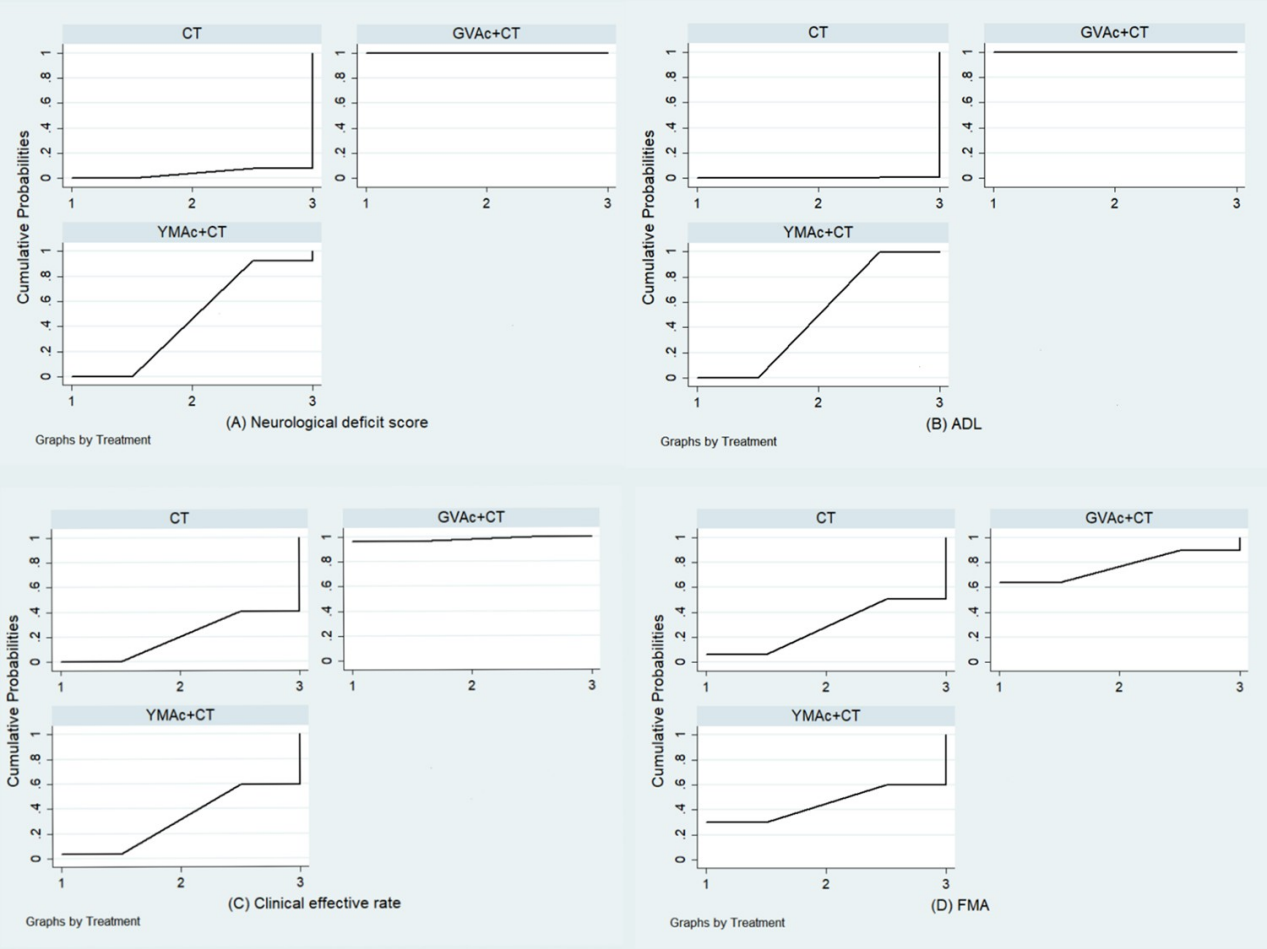
Comparative effectiveness between intervention categories: surface under the cumulative ranking curves (SUCRA) for (A): Neurological deficit score; (B): ADL; (C): Clinical effective rate; (D): FMA. **Note:** The x-axis is the possible level of each treatment (from the first to the worst according to different outcome indicators). The cumulative probability that each treatment is the best, second, and third best intervention is represented by the y axis. Consequently, each treatment method’s ranking is represented by the area under the curve. The likelihood that a treatment approach falls into the highest level or one of the highest levels increases as the value increases.

According to the result of ranking graph based on SUCRA, GVAc+CT was the most effective intervention to improve neurological deficit score in patients, with a SUCRA value of 99.9%, followed by 46.2% for YMAc+CT, and 3.9% for CT (Fig 7A).

#### ADL

According to the result of ranking graph based on SUCRA, GVAc+CT was the most effective intervention to improve ADL in patients. As shown in Fig 7B, the highest SUCRA value was 100% for GVAc+CT, followed by 49.7% for YMAc+CT, and 0.3% for CT (Fig 7B).

#### Clinical effective rate

According to the result of ranking graph based on SUCRA, GVAc+CT was the most effective intervention to improve clinical effective rate in patients, with a SUCRA value of 98.1%, followed by 31.5% for YM+C, and 20.3% for C (Fig 7C).

#### FMA

According to the result of ranking graph based on SUCRA, GVAc+CT was the most effective intervention to improve FMA in patients, with a SUCRA value of 76.6%, followed by 45% for YMAc+CT, and 28.5% for CT (Fig 7D).

### Heterogeneity analysis

When observing the basic characteristics of the included literature, there were differences in the use of electroacupuncture and non-electroacupuncture in different literatures, and the stimulation frequency used was also different, which might affect the stability of the results. Studies have shown [31] that the neurological deficit score is an objective basis for evaluating the overall stroke condition and prognosis of patients, which can be the most representative reflecting the clinical effect of acupuncture treatment. Therefore, we chose the neurological deficit score as the representative index.

**Fig 8:**
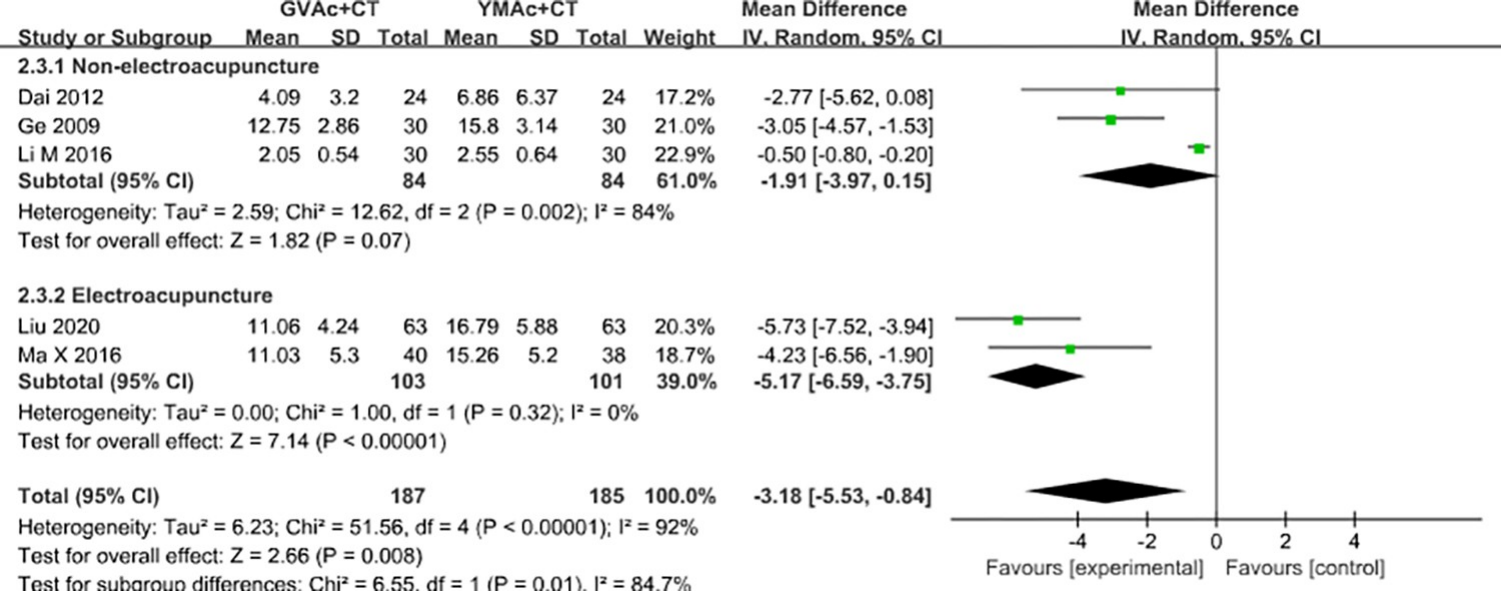
Forest plot of subgroup analysis with acupuncture manipulation as the criterion.

We divided the studies into the non-EA group and the EA group according to whether electricity was applied after acupuncture, and there was statistical heterogeneity among the studies (I^2^ = 94%, P<0.00001). The random-effects model was then used for meta-analysis. The combined effect was statistically significant (MD = -3.28, 95% = [-5.98, -0.58], P = 0.02). The results confirmed that compared with Yangming meridian acupuncture, governor vessel acupuncture could better reduce the neurological deficit score. Further subgroup analyses showed that: (1) Two studies described the use of non-EA, but the pooled effect size was not statistically significant (MD = -1.66, 95% = [-4.15, 0.83], P = 0.19), suggesting that there was no statistically significant difference in the therapeutic effect between governor vessel Acupuncture and Yangming meridian acupuncture. (2) Two studies reported the use of EA, and the results showed that acupuncture at governor vessel was significantly better than acupuncture at Yangming meridian in improving the degree of neurological deficit (MD = -5.17, 95% = [-6.59, -3.75], P<0.00001). (Fig 8). Based on the above results, we speculate that the use of EA may be a potential source of heterogeneity.

**Fig 9:**
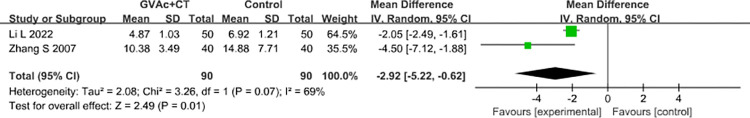
Forest plots depicting stimulus frequencies.

In addition, two studies described the stimulation frequency, and there was statistical heterogeneity between studies (I^2^ = 69%, P = 0.07). The results suggested that the governor vessel therapy could reduce the neurological deficit score more effectively than the conventional treatment, and the difference was statistically significant (MD = -2.92, 95% = [-5.22, -0.62], P = 0.01). (Fig 9). Therefore, we speculate that different stimulation frequencies may be a potential factor causing heterogeneity.

### Correction-compare funnel diagram

**Fig 10:**
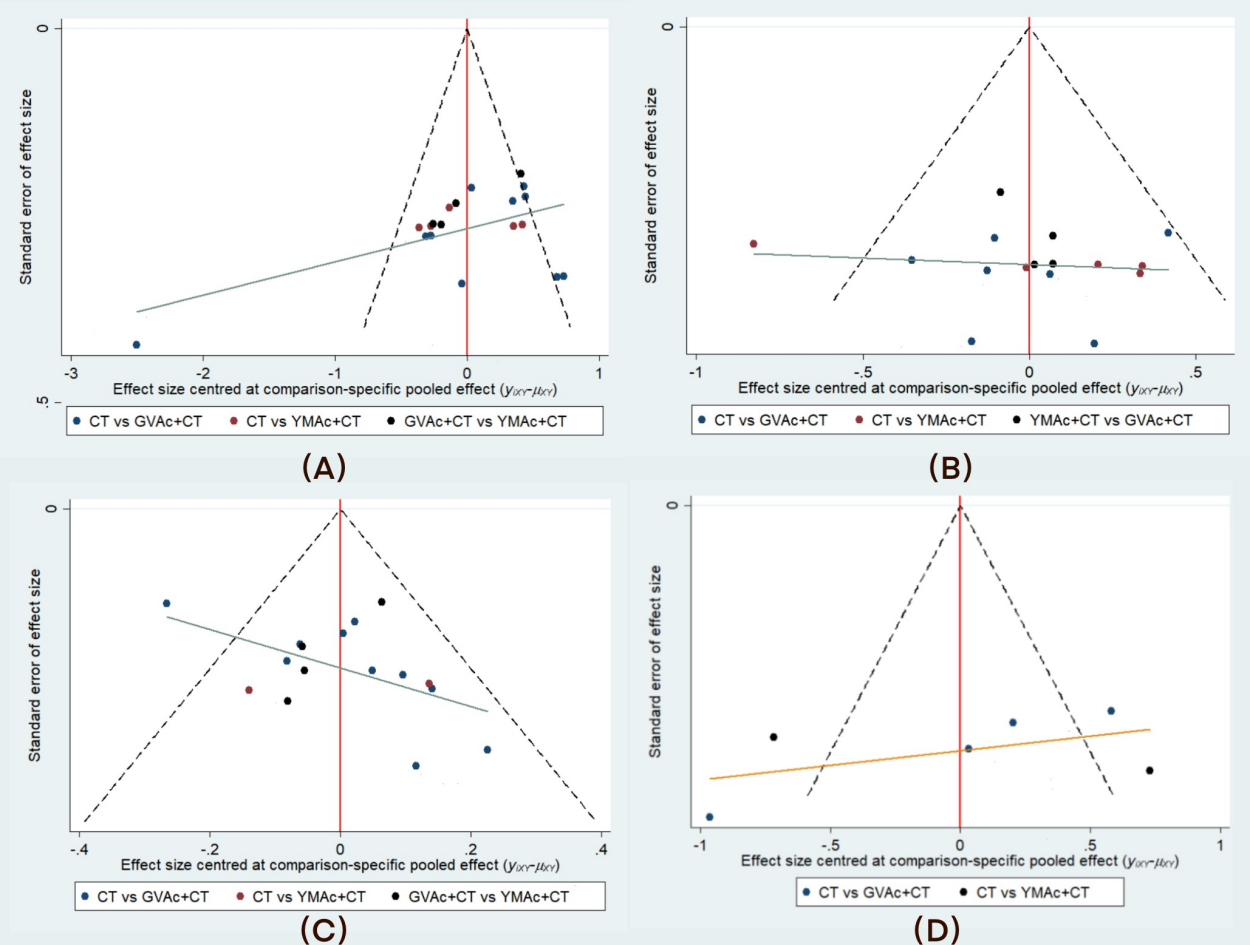
Correction-compare funnel diagram. **Note:** (A) Neurological deficit score, (B) ADL, (C) Clinical effective rate, (D) FMA.

We plotted comparative-corrected funnel diagram of the neurological deficit score, ADL, clinical effective rate and FMA using Stata 14.0 (Fig 10). The result showed that the studies were roughly in the central, and had good symmetry, suggesting that publication bias is unlikely.

### Adverse events

Adverse events were reported in 4 of the included studies. Because the occurrence of adverse reactions was inconsistent among the studies, only descriptive analysis was performed. 3 [16, 20, 26] of the included studies mentioned adverse events, including subcutaneous hematoma and stagnant needles, during treatment, all of which occasionally occurred after acupuncture treatment, and they were improved rapidly after active management. One study [19] referred to the follow-up of adverse events after treatment, and the results showed that the incidence of adverse events was much lower in the governor vessel acupuncture group than in the conventional treatment group, suggesting that the governor vessel acupuncture group has a better effect in improving the prognosis of patients with AIS. No other adverse events were observed in the included studies.

## Discussion

AIS is the second leading cause of death worldwide and the primary cause of permanent disability [32]. Extensive studies have proven that acupuncture intervention is an effective method to promoting nerve repair and improving functional dysfunction in patients with AIS [33]. There have not been any network meta-analysis studies comparing the effects of AIS acupuncture treatment based on meridians. In this network meta-analysis consisting of 17 RCTs we concluded that the efficacy of governor vessel acupuncture was the most significant in the treatment of AIS.

Our systematic review and network meta-analysis focused on the neurological deficit score ADL, clinical effective rate and FMA, The results showed that: (1) Regarding neurological deficit score, GVAc+CT exhibited superiority over YMAc+CT and CT (P < 0.05); (2) In terms of ADL, GVAc+CT dedmonstrated superiority to YMAc+CT and CT (P < 0.05). (3) In terms of the clinical efficacy, GVAc+CT showed superiority to CT (P < 0.05). The above results proved that governor vessel acupuncture has advantages in the treatment of AIS. The results of SUCRA ranking chart of each outcome indicator were as follows: "GVAc+CT" > "YMAc+CT" > "CT". In terms of improving ADL, the probability of GVAc+CT being the best therapy was 100%, showing an absolute advantage among the three therapies, suggesting that GVAc+CT was the most likely to be the most effective intervention for AIS. However, this result is still affected by the quality of evidence and the risk of bias.

Our evidence certainty assessment found that the included studies had some risk of bias, while the overall quality of evidence was generally low and very low. Due to the use of network meta-analysis, the evidence obtained from this study was indirect. Among them, the quality of evidence supports that GVAc+CT is superior to YMAc+CT and CT in reducing the degree of neurological deficit and improving ADL, even though there were some defects in the design of clinical RCTs included in the study. In terms of the clinical effective rate, the quality of evidence is very low in supporting that GVAc+CT is superior to YMAc+CT in improving the clinical effective rate. This may be related to the imprecision of research data and the small sample size. The quality of evidence between different interventions in enhancing FMA was very low, mainly due to the inconclusive evidence for this outcome measure, and there was little or no direct comparison evidence, suggesting that future relevant clinical RCT studies can further explore the efficacy of each therapy in enhancing FMA in patients with AIS.

We also conducted the heterogeneity analysis around the differences between the use of EA and non-EA, as well as the differences in stimulation frequency of acupuncture: (1) Four studies [18, 23, 24, 28] were divided into two groups according to whether EA was used or not. The results showed that the performance of the EA group was relatively homogeneous (I2 = 0%, P<0.00001), although the heterogeneity was decreased in the non-EA group, the difference between the two groups was not statistically significant (I2 = 90%, P = 0.19). Therefore, we speculated that the use of EA may be a potential source of heterogeneity. (2) The Meta-analysis showed that there was some heterogeneity between the two studies describing the stimulation frequency (I2 = 69%, P = 0.01), which may affect the heterogeneity of the results. We can speculate that the use of EA and stimulation frequency may affect the stability of the results, but whether it is the main factor affecting the heterogeneity needs to be discussed in more studies.

There are some limitations of this study: (1) The assessment of FMA outcome was limited by the absence of a direct comparison between GVAc + CT and YMAc + CT. Additionally, the small sample size available for analysis of FMA outcome might contribute to the uncertainty regarding the efficacy superiority of Governor Vessel acupuncture therapy to Yangming meridian acupuncture in improving FMA (P > 0.05). Which suggests that in the future, when treating AIS patients with severe limb motor deficits, we can add Yangming meridian acupuncture treatment, or carry out further research in this aspect. (2) The quality of evidence in this study was downgraded mainly due to limitations in study design, including issues related to blinding and allocation concealment as well as the imprecision of the data. (3) All eligible studies included in the analysis were from China and the results were based on the Chinese population, but may not generalize well to other races.

## Conclusion

This study confirmed that the efficacy of governor vessel acupuncture is better than Yangming meridian acupuncture for the treatment of AIS. However, due to the lack of definitive evidence on FMA outcome indicators and the low quality of evidence on improving FMA in patients with AIS, we were unable to conclude the true effect of governor vessel acupuncture in improving FMA in patients with AIS. Further investigations are needed to demonstrate the efficacy of the governor vessel acupuncture in the treatment of AIS by carrying out different multi-ethnic, large-sample, clinical randomized controlled studies in the future.

## Supporting information

S1 File PRISMA NMA checklist. (PDF)

S2 File Search formulas. (PDF)

## List of abbreviations used

AIS: Acute ischemic stroke
SUCRA: surface under the cumulative ranking curve
GV1: Changqiang
GV28: Yinjiao
LI1: Shangyang
LI20: Yingxiang
ST1: Chengqi
ST12: Quepen
ST30: Qichong
ST44: Lidui
ADL: Activities of daily living
FMA: Fugl-meyer motor function evaluation
RCTs: randomized controlled trials
SMD: standardized mean difference
RR: relative risk
h: hour
d: day
w: week

## References

[1] Walter K. What Is Acute Ischemic Stroke? JAMA. 2022 Mar;327(9):885. doi: 10.1001/jama.2022.1420 35230392

[2] Dirnagl U. Pathobiology of injury after stroke: the neurovascular unit and beyond. Ann N Y Acad Sci. 2012 Sep;1268:21–5. doi: 10.1111/j.1749-6632.2012.06691.x 22994217

[3] Mu JD, Ma LX, Zhang Z, Qian X, Zhang QY, Ma LH, et al. The factors affecting neurogenesis after stroke and the role of acupuncture. Front Neurol. 2023 Jan;14:1082625. doi: 10.3389/fneur.2023.1082625 36741282 PMC9895425

[4] Chavez LM, Huang SS, MacDonald I, Lin JG, Lee YC, Chen YH. Mechanisms of Acupuncture Therapy in Ischemic Stroke Rehabilitation: A Literature Review of Basic Studies. Int J Mol Sci. 2017 Oct;18(11):2270. doi: 10.3390/ijms18112270 29143805 PMC5713240

[5] Kou YF, Cai EL, Zhang W, Wu YM. Study on the protective effect of electroacupuncture intervention time on brain after cerebral ischemia reperfusion in rats. Journal of Guizhou University of Traditional Chinese Medicine. 2014;36(04):39–41. doi: 10.3969/j.issn.1002-1108.2014.04.016

[6] World Health Organization. Acupuncture: review and analysis of reports on controlled clinical trials. Parkinsonism & Related Disorders. 2003. doi: 10.1016/S1353-8020(11)70706-4

[7] Jin ZQ, Cheng JS. Effects of acupuncture at different acupoints on cortical somatosensory evoked potential and infarct volume in rats with transient focal cerebral ischemia. Shanghai Journal of Acupuncture and Moxibustion. 1998;39–41. doi: 10.13460 j.issn.1005

[8] Zhou F, Guo JC, Cheng J, Wu G, Sun J, Xia Y. Electroacupuncture and Brain Protection against Cerebral Ischemia: Specific Effects of Acupoints. Evid Based Complement Alternat Med. 2013; 2013:804397. doi: 10.1155/2013/804397 23737846 PMC3666307

[9] Wang SL. Effect of acupuncture on the mRNA expression of MAP-2 and NF-L in the ischemic penumbra of rats undergoing cerebral ischemia and reperfusion. M.Sc. Thesis, Nanjing University of Chinese Medicine. 2012. Available from: 10.7666/d.y2124025

[10] Pan J, Chen WS, Chen C, Zhang W. Effects of Electric-Acupuncture at Du-Meridian on MCAO Rats Infarct Volume and NGF in Brain Tissue. Chinese Archives of Traditional Chinese Medicine. 2017;(03),541–543. doi: 10.13193/j.issn.1673-7717.2017.03.007

[11] Chen GM, Huang CY, Liu YY, Zhang ZP, Qi XQ, Shi PY, et al. Efficacy and safety of grain moxibustion in hemiplegia: A systematic review and meta-analysis protocol. Medicine (Baltimore). 2019 Apr;98(17):e15215. doi: 10.1097/MD.0000000000015215 31027068 PMC6831348

[12] Neurology Branch of Chinese Medical Association, Cerebrovascular Disease Group of Neurology Branch of Chinese Medical Association. Guidelines for the diagnosis and treatment of acute ischemic stroke in China 2014.Chinese Journal of Neurology. 2015,48(4): 246–257. doi: 10.3760/cma.j.issn.1006-7876.2015.04.002

[13] Higgins JPT, Altman DG, Gøtzsche PC, Jüni P, Moher D, Oxman AD, et al. The Cochrane Collaboration’s tool for assessing risk of bias in randomised trials. BMJ. 2011, 34:d5928. doi: 10.1136/bmj.d5928 22008217 PMC3196245

[14] Wei CY, Ma YB, Li XH. Application effect of Tongdu Tiaoshen acupuncture in patients with acute cerebral infarction hemiplegia. Lingnan Journal of Emergency Medicine,2023,28(01):53–55.

[15] Li LY, Qiao J, Ding HY. Effect of acupuncture method in the treatment of patients with acute cerebral infarction. Medical Journal of Chinese People’s Health. 2022;34(11):111–113. doi: 10.3969/j.issn.1672-0369.2022.11.035

[16] Ma C, Li J, Wang XN, Chang YX. Clinical study on effect of Shengyang Tongdu acupuncture on electroencephalography topography, neurovascular unit function and heart ratevariability in patients with cerebral infarction. Shanghai Journal of Traditional Chinese Medicine. 2021;55(09):50–54. doi: 10.16305/j.1007-1334.2021.1912090

[17] Wang XW, Tan F, Li DD, Zhan J, Fang MF, Li M, et al. Influence of Electroacupuncture on Motor Dysfunction and Corticospinal TractInjury After Acute lschemic Stroke. Shanghai Journal of Acupuncture and Moxibustion. 2021;40(04):426–430. doi: 10.13460/j.issn.1005-0957.2021.04.0426

[18] Liu ZY. Analysis of the effect of acute cerebral infarction with hyperhomocysteinemia by transfer-through-governor injection. Smart Healthcare. 2020;6:153–4,7. doi: 10.19335/j.cnki.2096-1219.2020.05.066

[19] Hua HB, Qian FW, Zheng WZ. Effect of acupuncture and moxibustion combined with tongdu therapy on ischemic stroke and its influence on prognosis. Shanghai Journal of Acupuncture and Moxibustion. 2019;38:1087–92. doi: 10.13460/j.issn.1005-0957.2019.10.1087

[20] Li YW. Study on the effect and curative effect of Tongdu Shenregulating acupuncture on ischemic penumbra in patients with acute ischemic stroke. M.Sc. Thesis, Anhui University of Traditional Chinese Medicine. 2018.

[21] Zhang L, Zhang GQ, Zhu LL, Han W. Clinical observation on the treatment of wind-phlegm-Stasis acute cerebral infarction by Tongdu-Tiaoshen acupuncture. Journal of Anhui Traditional Chinese Medical College. 2017;36:59–62. doi: 10.3969/j.issn.2095-7246.2017.04.018

[22] Cai J, Tang CZ, Zhu GQ. Clinical study on acupuncture Dumai point in the treatment of acute cerebral infarction. Asia-Pacific Traditional Medicine. 2016;12:116–7. doi: 10.11954/ytctyy.201603054

[23] Li MX, Sha YP. The effect of ’ Xingnao Kaiqiao ’ acupuncture on acute ischemic stroke and its effect on MMP-9. Journal of Clinical Acupuncture and Moxibustion. 2016;32(12):17–19.

[24] Ma XM, Li JJ, Yan B, Liu YF, Zou P, Huang RC. Clinical study on the treatment of acute cerebral infarction complicated with hyperhomocysteine by transfer-general acupuncture. Journal of Shaanxi College of Traditional Chinese Medicine. 2016;39:50–3,102. doi: 10.13424/j.cnki.jsctcm.2016.04.018

[25] Yu FF, Clinical study of combining self-rehabilitation training for the treatment of acute stroke hemiplegia. M.Sc. Thesis, Guangzhou University of Chinese Medicine. 2014; 49.

[26] Tan JC, Ding WT, Ge L. Effect of early acupuncture on 46 cases of acute cerebral infarction complicated with type 2 diabetes mellitus. Hebei Traditional Chinese Medicine,2013,35(5):722–724.

[27] Huang T, Li GX.The effect of electroacupuncture on the expression of CD62p and D-Dimer and the scores of ADL and NIHSS in patients with acute cerebral infarction. Lishizhen Medicine and Materia Medica Research. 2012;23(10):2665–2667. doi: 10.3969/j.issn.1008-0805.2012.10.128

[28] Ge CH. The effect of acupuncture on CRP and D-dimer in patients with acute cerebral infarction and its curative effect. M.Sc. Thesis, Guizhou: Guiyang College of Traditional Chinese Medicine. 2009. Available from: 10.7666/d.y1561180

[29] Zhang X. Effect of electroacupuncture of Yangming meridian acupoint on the expression of PAC-1 and CD62p in patients with acute cerebral infarction. M.Sc. Thesis, Guangzhou University of Chinese Medicine. 2008.

[30] Zhang SJ, Chen DF, Lai Z. Clinical observation of acupuncture Dumai point in the treatment of acute cerebral infarction. Journal of Changchun University of Chinese Medicine. 2007;23:57–8. doi: 10.3969/j.issn.1007-4813.2007.05.037

[31] Dong Q, Wu DC, Lv CZ. Compare Analysis of the Neurological Functional Deficit Scales in 193 Patients with Acute Stroke. Chinese Journal of Clinical Neurosciences. 2000. doi: 10.3969/j.issn.1008-0678.2000.03.011

[32] Farina M, Vieira LE, Buttari B, Profumo E, Saso L. The Nrf2 Pathway in Ischemic Stroke: A Review. Molecules (Basel, Switzerland). 2021;26(16),5001. doi: 10.3390/molecules26165001 34443584 PMC8399750

[33] Chang QY, Lin YW, Hsieh CL. Acupuncture and neuroregeneration in ischemic stroke. Neural regeneration research. 2018;13,573–583. doi: 10.4103/1673-5374.230272 29722298 PMC5950656

