# Supplementary material for: Effectiveness of acupuncture in the governor vessel and Yangming meridian for the treatment of acute ischemic stroke: A systematic review and network meta-analysis
Source: PLoS One. 2024 Apr 16;19(4):e0300242. doi: 10.1371/journal.pone.0300242 (PMC11021022; doi:10.1371/journal.pone.0300242)
Supplement: S2 File — (PDF) [file pone.0300242.s002.pdf]

1.VIP

Search strategy:

M=(急性缺血性中风 OR 急性缺血性脑中风 OR 急性脑梗死 OR 急性脑梗塞 OR 急性脑栓塞 OR 急性缺血性脑卒中 OR 急性脑缺血 OR 急性缺血性卒中 OR 急性缺血性脑血管病 OR 急性缺血性脑血管意外 OR 急性脑血栓 or 缺血性中风急性 OR 缺血性脑中风急性 OR 脑梗死急性 OR 脑梗塞急性 OR 脑栓塞急性 OR 缺血性脑卒中急性 OR 脑缺血急性 OR 缺血性卒中急性 OR 缺血性脑血管病急性 OR 缺血性脑血管意外急性 OR 脑血栓急性) AND (阳明 OR 阳明经 OR 足阳明胃经 OR 手阳明大肠经 OR 督 OR 督脉 OR 督经) AND (针 OR 针灸 OR 针刺 OR 毫针)

2.CNKI

Search strategy:

SU%=('急性缺血性中风' + '急性脑梗死' + '急性脑梗塞' + '急性脑栓塞' + '急性缺血性脑卒中' + '急性脑缺血' + '急性缺血性卒中' + '急性缺血性脑血管病' + '急性缺血性脑血管意外' + '急性脑血栓' + '缺血性中风急性' + '缺血性脑中风急性' + '脑梗死急性' + '脑梗塞急性' + '脑栓塞急性' + '缺血性脑卒中急性' + '脑缺血急性' + '缺血性卒中急性' + '缺血性脑血管病急性' + '缺血性脑血管意外急性' + '脑血栓急性') AND SU%=('阳明' + '阳明经' + '足阳明胃经' + '手阳明大肠经' + '督' + '督脉' + '督经') AND FT=('针' + '毫针' + '针刺' + '针灸')

3.WANFANG

Search strategy:

主题:(“急性缺血性中风” or “急性脑梗死” or “急性脑梗塞” or “急性脑栓塞” or “急性缺血性脑卒中” or “急性脑缺血” or “急性缺血性卒中” or “急性缺血性脑血管病” or “急性缺血性脑血管意外” or “急性脑血栓” or “缺血性中风急性” or “缺血性脑中风急性” or “脑梗死急性” or “脑梗塞急性” or “脑栓塞急性” or “缺血性脑卒中急性” or “脑缺血急性” or “缺血性卒中急性” or “缺血性脑血管病急性” or “缺血性脑血管意外急性” or “脑血栓急性” ） and 主题:(“阳明” or “阳明经” or “足阳明胃经” or “手阳明大肠经”or “督” or “督脉” or “督经”) and 主题:(“针” or “针灸” or “针刺”or “毫针”)

4. Sinomed

Search strategy:

("针"[全部字段:智能] OR "针灸"[全部字段:智能] OR "针刺"[全部字段:智能] OR "毫针"[全部字段:智能]) AND("急性缺血性中风"[标题:智能] OR "急性缺血性脑中风"[标题:智能] OR "急性脑梗死"[标题:智能] OR "急性脑梗塞"[标题:智能] OR "急性脑栓塞"[标题:智能] OR "急性缺血性脑卒中"[标题:智能] OR "急性脑缺血"[标题:智能] OR "急性缺血性卒中"[标题:智能] OR "急性缺血性脑血管病"[标题:智能] OR "急性缺血性脑血管意外"[标题:智能] OR "急性脑血栓 or 缺血性中风急性"[标题:智能] OR "缺血性脑中风急性"[标题:智能] OR "脑梗死急性"[标题:智

能] OR "脑梗塞急性"[标题:智能] OR "脑栓塞急性"[标题:智能] OR "缺血性脑卒中急性"[标题:智能] OR "脑缺血急性"[标题:智能] OR "缺血性卒中急性"[标题:智能] OR "缺血性脑血管病急性"[标题:智能] OR "缺血性脑血管意外急性"[标题:智能] OR "脑血栓急性"[标题:智能]) AND("阳明"[全部字段:智能] OR "阳明经"[全部字段:智能] OR "足阳明胃经"[全部字段:智能] OR "手阳明大肠经"[全部字段:智能] OR "督"[全部字段:智能] OR "督脉"[全部字段:智能] OR "督经"[全部字段:智能])

5. Web of Science

Search strategy:

((TS=(Acute ischemic stroke OR Acute brain Infarction OR Acute Ischemia Apoplexy OR Acute cerebral infarction OR Acute cerebral embolism OR Acute Ischemic Apoplexy OR Acute brain Ischemia OR Acute Ischemic Encephalopathies OR AIS OR ACI)) AND TS=(governor vessel OR governor meridian OR du meridian OR yangming meridian)) AND TS=(acupuncture OR electropuncture)

6. Pub Med

Search strategy:

((((((((((Acute ischemic stroke[Title/Abstract]) OR (Acute brain Infarction[Title/Abstract])) OR (Acute Ischemia Apoplexy[Title/Abstract])) OR (Acute cerebral infarction[Title/Abstract])) OR (Acute cerebral embolism[Title/Abstract])) OR (Acute Ischemic Apoplexy[Title/Abstract])) OR

(Acute brain Ischemia[Title/Abstract])) OR (Acute Ischemic Encephalopathies[Title/Abstract])) OR (AIS[Title/Abstract])) OR (ACI[Title/Abstract])) AND ((((governor vessel[Title/Abstract]) OR (governor meridian[Title/Abstract])) OR (du meridian[Title/Abstract])) OR (yangming meridian[Title/Abstract]))) AND (acupuncture[Title/Abstract] OR electropuncture[Title/Abstract])

7. Cochrane Library

Search strategy:

(Title Abstract Keyword: Acute ischemic stroke or Acute brain Infarction or Acute Ischemia Apoplexy or Acute cerebral infarction or Acute cerebral embolism or Acute Ischemic Apoplexy or Acute brain lschemia or Acute Ischemic Encephalopathies or AIS or ACI) AND (governor vessel or governor meridian or du meridian or yangming meridian) AND (acupuncture or electropuncture)

8. Embase

Search strategy:

((Acute ischemic stroke or Acute brain Infarction or Acute Ischemia Apoplexy or Acute cerebral infarction or Acute cerebral embolism or Acute Ischemic Apoplexy or Acute brain lschemia or Acute Ischemic Encephalopathies or AIS or ACI) and (governor vessel or governor meridian or du meridian or yangming meridian) and acupuncture or electropuncture).ti.
